# Effects of a lifestyle intervention in routine care on prenatal physical activity – findings from the cluster-randomised GeliS trial

**DOI:** 10.1186/s12884-019-2553-7

**Published:** 2019-11-11

**Authors:** Julia Hoffmann, Julia Günther, Kristina Geyer, Lynne Stecher, Kathrin Rauh, Julia Kunath, Dorothy Meyer, Christina Sitzberger, Monika Spies, Eva Rosenfeld, Luzia Kick, Renate Oberhoffer, Hans Hauner

**Affiliations:** 1 Else Kröner-Fresenius-Centre for Nutritional Medicine, Klinikum rechts der Isar, Technical University of Munich, Georg-Brauchle-Ring 62, 80992 Munich, Germany; 2 Competence Centre for Nutrition (KErn), Am Gereuth 4, 85354 Freising, Germany; 3 Institute of Preventive Pediatrics, Technical University Munich, Georg-Brauchle-Ring 62, 80992 Munich, Germany; 4 Department of Pediatric Cardiology and Congenital Heart Defects, German Heart Centre, Lazarettstraße 36, 80636 Munich, Germany

**Keywords:** Physical activity, Exercise, Lifestyle intervention, Pregnancy, Gestational weight gain (GWG), Obesity prevention, Routine care

## Abstract

**Background:**

Excessive gestational weight gain (GWG) is associated with an increased risk of pregnancy and obstetric complications. The “healthy living in pregnancy” (GeliS) study was performed in a routine care setting with the aim of limiting excessive GWG. The purpose of this secondary analysis is to evaluate the effect of the intervention on physical activity (PA) behaviour and to assess the impact of PA intensities on GWG.

**Methods:**

The cluster-randomised, multicentre GeliS trial was performed in a routine care setting alongside scheduled prenatal visits. Pregnant women with a pre-pregnancy BMI between 18.5 and 40.0 kg/m^2^ were either assigned to the control group receiving usual care or to the intervention group. Participants in the intervention group attended three antenatal counselling sessions on diet and PA and one additional postpartum session. Data on PA behaviour were collected twice, before the end of the 12th (baseline) and after the 29th week of gestation using the Pregnancy Physical Activity Questionnaire.

**Results:**

PA data were available for 1061 (93%) participants in the intervention and 1040 (93%) in the control group. Women in the intervention group reported significant improvements in the levels of total PA (*p* < 0.001), total PA of light intensity and above (*p* < 0.001), moderate-intensity (*p* = 0.024) and vigorous-intensity activities (*p* = 0.002) as well as sport activities (*p* < 0.001) in late pregnancy compared to the control group. The proportion of women meeting the international PA recommendations in late pregnancy was significantly higher in the intervention (64%) versus the control group (49%, *p* < 0.001). Activities of light-intensity and above (*p* = 0.006), light-intensity (*p* = 0.002) and vigorous-intensity (*p* = 0.014) in late pregnancy were inversely associated with total GWG.

**Conclusion:**

We found significant evidence of improvements in the PA pattern of pregnant women receiving lifestyle counselling within the framework of routine care. Most PA intensities were inversely associated with total GWG which indicates that PA across different intensities should be promoted.

**Trial registration:**

NCT01958307, ClinicalTrials.gov, retrospectively registered 9 October, 2013.

## Background

Excessive gestational weight gain (GWG) is associated with several pregnancy and foetal complications such as gestational diabetes mellitus (GDM), caesarean section, preterm delivery and high birth weight [1–6]. Moreover, excessive GWG may influence maternal and infant long-term health. Research suggests that GWG is not only a determinant of maternal postpartum weight retention, but also increases the risk of obesity in both mother and child [7–13]. The U.S. Institute of Medicine (IOM) proposed guidelines to define excessive GWG according to a woman’s pre-pregnancy Body-Mass-Index (BMI) [14]. In Western countries, there is a trend towards an increase in the rate of excessive GWG [15]. In Germany, more than 40% of pregnant women exceed the recommended IOM thresholds [16]. Next to dietary behaviour, prenatal physical activity (PA) seems to be a major determinant of GWG. Apart from associations with GWG, prenatal PA was shown to beneficially influence several physiological functions for instance in the cardiovascular and pulmonary systems [17, 18], and to lower the risk for pregnancy-induced complications such as GDM, preeclampsia and caesarean section [19–22]. Moreover, PA improves a woman’s psychological well-being and quality of life in general as well as during pregnancy and decreases the risk for anxiety and depressive symptoms including postpartum depression [23–27]. The evidence outlined above clearly demonstrates that PA plays a fundamental role with respect to a woman’s health status during pregnancy and in the postpartum period. However, only a minor proportion of pregnant women meets current PA recommendations [18, 28, 29]. Moreover, PA often declines over the course of pregnancy [30, 31]. This emphasises the need to develop interventions that address the prenatal lifestyle and include strategies to improve PA behaviour in order to reduce both excessive GWG and pregnancy-induced complications.

In the last decade, various lifestyle interventions focusing on dietary and PA behaviour have been initiated to prevent excessive GWG and to minimise resulting health complications for mothers and their infants. Most randomised-controlled trials (RCTs) showed rather modest effects in the prevention of excessive GWG [32–35] and a recent meta-analysis suggested a decrease of GWG by − 0.70 kg in women receiving lifestyle advice [36]. However, RCTs differed in their design, outcomes measures, procedures, study population, sample size as well as mode and intensity of the intervention. Only a small number of RCTs were conducted outside academic settings and implemented in routine prenatal care [37–39]. With respect to the overall increasing rates of excessive GWG in all BMI categories and concomitant adverse health outcomes, it remains a challenge to establish effective and efficient interventions in “real-life” settings.

In this context, the FeLIPO trial (“Feasibility of a Lifestyle-Intervention in Pregnancy to Optimize maternal weight development”) was conducted in a routine care setting with the aim of reducing the number of pregnant women exceeding the IOM recommendations. The intervention consisted of two prenatal counselling sessions focusing on diet and PA and led to beneficial effects on the proportion of women with excessive GWG and on some lifestyle factors [40].

The FeLIPO trial encouraged us to offer a lifestyle intervention programme within the framework of the well-established German prenatal care system, the “Gesund leben in der Schwangerschaft”/“healthy living in pregnancy” (GeliS) trial [41]. By implementing an intervention under real-life conditions, the primary aim of the GeliS trial was to reduce the proportion of women with excessive GWG. The effect of the GeliS intervention on excessive GWG has been published recently [42]. Furthermore, the trial endeavoured to improve women’s prenatal lifestyle and to maintain or even increase their PA in accordance with national and international PA recommendations [43, 44]. This secondary analysis aims to investigate the PA behaviour of women enrolled in the GeliS trial and its influence on GWG.

## Methods

### Objectives

The primary outcome of the GeliS study was to reduce the proportion of participating women with excessive GWG according to the IOM recommendations [41]. Primary and some secondary outcomes have been published recently [42, 45–47].

This secondary analysis mainly aimed to investigate the effect of the GeliS lifestyle intervention on antenatal PA behaviour by exploring differences in PA of women receiving the GeliS intervention (IV) compared to women receiving usual care only (C) and more specifically, to identify factors that could have influenced prenatal PA behaviour. Additionally, we were interested in investigating the effect of PA intensities on GWG in the entire cohort. For these analyses, the intervention and control groups were pooled to report cohort data.

### The GeliS study: design and setting

The design of the GeliS public health project has been described previously [41]. In brief, it was a prospective, multicentre, cluster-randomised, controlled, open intervention trial conducted alongside prenatal routine care in five administrative regions of Bavaria (Germany). Within each administrative region, pairwise randomisation was conducted by randomly matching two districts (cluster) per region according to birth figures, sociodemographic and geographic criteria which resulted in one control district and one intervention district per region. Within these districts, the study was conducted in gynaecological and midwifery practices which represent “real-life” settings of routine prenatal care in Germany. The study was performed in accordance with current local regulatory requirements and according to the declaration of Helsinki. The study protocol was approved by the Ethics Committee of the Technical University of Munich and is registered in the ClinicalTrials.gov Protocol Registration System (NCT01958307).

### Participants

Between 2013 and 2015, medical personnel at 71 participating gynaecological and midwifery practices (39 in the intervention regions and 32 in the control regions) in both urban and rural regions recruited the participants. These practices varied in terms of the number of doctors, medical personnel as well as the number of participants that were recruited. Women were eligible if they had 1) a pre-pregnancy BMI between ≥ 18.5 kg/m^2^ and ≤ 40.0 kg/m^2^, 2) a singleton pregnancy, 3) age between 18 and 43 years, 4) sufficient German language skills and 5) stage of pregnancy before the end of the 12th week of gestation. All women gave their written informed consent for participation. As described in the study protocol [41], women with severe pre-existing diseases, multiple or complicated pregnancies were excluded from study participation. Reasons for drop-out during the course of the trial included miscarriage or late loss of pregnancy, terminations, pregnancy complications which interfered with the intervention and maternal death.

### Lifestyle intervention

Participants in the control group (C) attended routine prenatal care and obtained general information on a healthy prenatal lifestyle in the form of a flyer. Participants in the intervention group (IV) additionally received a comprehensive lifestyle intervention programme alongside prenatal visits that consisted of three face-to-face counselling sessions during pregnancy (12th–16th, 16th–20th, and 30th–34th week of gestation) and one after delivery at 6th–8th week postpartum, each lasting 30–45 min. Counselling sessions were given by previously trained medical personnel, midwives or gynaecologists. Within the counselling sessions, women were informed about an adequate GWG according to the IOM recommendations [14] and were encouraged to weekly monitor their weight gain by means of weight gain charts. Moreover, a healthy diet and an appropriate PA behaviour were addressed in accordance with national and international recommendations [43, 44]. Women were informed about the beneficial effects of prenatal PA on GWG as well as on physiological and psychological well-being. They were motivated to engage in at least 30 min in moderate-intensity PA on most days and to maintain or increase their level of daily routine activity. They were advised to perform low-impact endurance exercises such as swimming, walking, cycling or aquatic exercise instead of weight-bearing sports. Furthermore, they were provided with a pedometer as a self-motivating tool and brochures including examples of adequate exercise as well as a list of prenatal physical exercise programmes in their vicinity. Additionally, counsellors assessed the participant’s PA behaviour by means of the baseline questionnaire and provided individualised suggestions for improvement, focusing mainly on specific changes in the woman’s daily routine. Further details about the counselling content have been described in detail previously [41].

### Data collection

Baseline characteristics were collected using a screening questionnaire at the time of recruitment. Pre-pregnancy BMI was calculated on the basis of self-reported weight. GWG was defined as the difference between the latest measured weight at the last prenatal visit and the first measured weight at the first prenatal visit, both measured in medical practices. Maternal weight and health parameters were retrieved from the routinely used maternity records.

Prenatal PA behaviour was assessed at two time points during pregnancy (T0: baseline assessment before the end of the 12th week of gestation; T1: after the 29th week of gestation) using the validated Pregnancy Physical Activity Questionnaire (PPAQ) [48]. The questionnaire was slightly adapted to German habits. The question asking for time spent sitting on a lawnmower was not included, as these types of lawnmowers are rarely used in Germany. The questionnaire was completed by participants without supervision. The PPAQ asked participants to estimate the time spent during the past month in 32 activities. In two open-ended questions, participants had the option to report activities that were not listed within the remaining questions. The number of hours spent at each activity was multiplied by its intensity (metabolic equivalent of task, MET) provided by the calculation sheet of the PPAQ [49] and summed up to obtain a measure of average weekly energy expenditure in MET-h/week. The 2011 Compendium of Physical Activities [50] was used to assign the corresponding MET values to reported open-ended activities. Thereby, total PA and “Total PA of Light Intensity and Above” (TALIA) in MET-h/week were estimated. Moreover, the PPAQ allowed the classification of average weekly energy expenditure based on activity “type” and activity “intensity”. In the category “type”, activities were grouped into household activity, occupational activity, sports/exercise, transportation and inactivity. In the category “intensity”, activity intensities were defined as “sedentary” (MET < 1.5), “light” (MET ≥ 1.5 and < 3.0), “moderate” (MET ≥ 3.0 and ≤ 6.0), or “vigorous” activities (MET > 6.0). As done by others [7], questionnaires were excluded from the analysis due to over-reporting if the total number of hours reported in the PPAQ exceeded the total number of hours per week. If women reported spending more than 12 h per day for 7 days per week in occupational activity, they were classified as over-reporter in the category of occupational activity. PA was dichotomised in order to verify whether women met the national and international PA recommendations [43, 44]. As done by others [51] and recommended by the PPAQ developer (personal communication), a threshold of ≥ 7.5 MET-h/week in sport activities of moderate intensity was set for meeting the recommendations.

### Statistical analysis

A power calculation was performed based on the primary study outcome excessive GWG and was described elsewhere [41]. Statistical analyses were performed using SPSS software (IBM SPSS Statistics for Windows, version 24.0, IBM Corp, Armonk, NY, USA). Baseline characteristics are presented as mean and standard deviation (SD) or proportions if appropriate. PA behaviour (intensities and types) is presented in mean MET-h/week.

Due to the cluster-randomised design, linear regression models fit with generalised estimating equations (GEE) were applied to compare the PA intensities and types in late pregnancy (T1) between groups [52]. Unadjusted as well as models adjusted for pre-pregnancy BMI category, parity, age and baseline PA (T0) were fit. Group differences in the dichotomised variable “meeting the recommendations” were estimated by means of logistic regression models fit with GEEs and adjusted for the same covariates. To assess the time effect, the change in PA over the course of pregnancy, unadjusted linear mixed models for repeated measures and models adjusted for pre-pregnancy BMI category, parity and age were used. Subgroup analyses at T1 according to pre-pregnancy BMI category, different age categories and educational level were performed on the basis of TALIA using GEEs. By means of GEEs, the overall impact of pre-pregnancy BMI category, age categories and educational level on TALIA at both time points was assessed as well as potential interactions with group assignment at T1.

To assess the impact of prenatal PA on GWG, the intervention and control groups were pooled to form one cohort. Total GWG was associated with PA intensities by means of generalised linear regression models, controlling for pre-pregnancy BMI category, parity, age and group assignment as confounding factors. The effect of a change by 10 MET-h/week on total GWG was estimated.

In all analyses, a *p*-value below 0.05 was considered as statistically significant. PA analyses included all participants, without those who dropped out before delivery due to miscarriages or late loss of pregnancy, terminations, pregnancy complications interfering with the intervention and maternal deaths. As defined a priori [42], analyses relating to GWG or excessive GWG were conducted as complete-case analyses considering all participants with available GWG data except from those with preterm delivery (< 37th week of gestation). In addition, participants were excluded from single calculations of intensities or types of PA if one or more answers in the corresponding category were missing.

## Results

### Participant flow and baseline characteristics

**Fig. 1:**
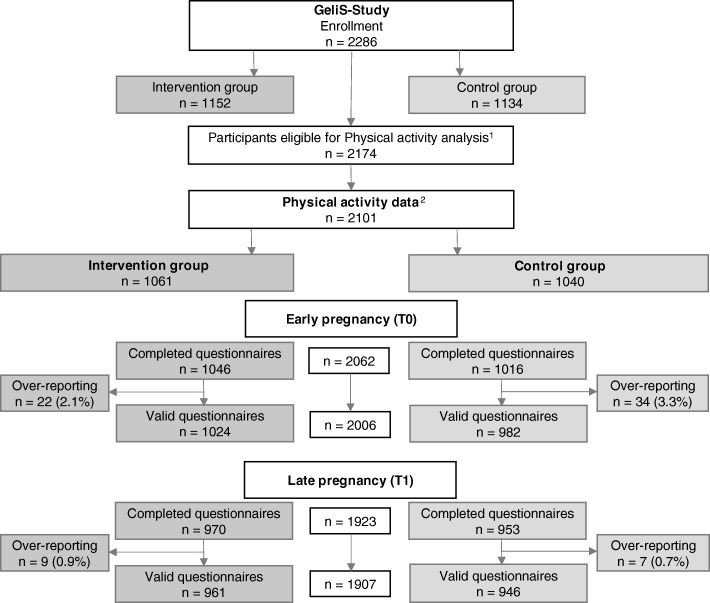
Participant flow in physical activity analysis. ^1^Excluding women who were not eligible when reassessed and women with miscarriages, late loss of pregnancy, terminations, pregnancy complications that interfere with the intervention and maternal deaths (*n* = 112). ^2^Women who provided PA data at T0 or T1. T0: Assessment before the end of the 12th week of gestation. T1: Assessment after the 29th week of gestation

In the GeliS study, 2286 participants were enrolled (IV: *n* = 1152; C: *n* = 1134) (Fig. 1). Among them, 53 participants of the IV and 59 participants of the C were either not eligible when reassessed or dropped out during the course of pregnancy and were thus not eligible for PA analyses. Among the 2174 study participants potentially eligible for PA analyses, 2101 provided PA data (IV: *n* = 1061; C: *n* = 1040). For the assessment of PA in early pregnancy (T0), *n* = 22 questionnaires of the IV and *n* = 34 of the C were excluded due to over-reporting which resulted in a total of 2006 valid questionnaires (IV: *n* = 1024; C: *n* = 982). For the same reasons, *n* = 9 questionnaires of the IV and *n* = 7 questionnaires of the C were excluded in late pregnancy resulting in a total of 1907 valid questionnaires (IV: *n* = 961; C: *n* = 946).

Table 1 shows baseline characteristics of participants included in the PA analysis. Mean self-reported pre-pregnancy weight and BMI were comparable in both groups (IV: 68.4 kg and 24.4 kg/m^2^; C: 67.9 kg and 24.3 kg/m^2^). In total, 65.3% of women had a normal weight, 22.8% overweight and 12.0% obesity. In the IV, more women were nulliparous (62.2%) compared to the C (53.6%). Maternal age and educational level were comparable between the two groups.

**Table 1:** Baseline characteristics of participants with available physical activity data

	**Intervention (*n* = 1061)**	**Control (*n* = 1040)**	**Total (*n* = 2101)**
Pre-pregnancy age (years)^a^	30.1 ± 4.3	30.3 ± 4.6	30.2 ± 4.5
Pre-pregnancy weight (kg)^a^	68.4 ± 13.1	67.9 ± 13.7	68.2 ± 13.4
Pre-pregnancy BMI (kg/m^2^)^a^	24.4 ± 4.4	24.3 ± 4.6	24.4 ± 4.5
Pre-pregnancy BMI category (n (%))			
BMI 18.5–24.9 kg/m^2^	684/1061 (64.5%)	687/1040 (66.1%)	1371/2101 (65.3%)
BMI 25.0–29.9 kg/m^2^	253/1061 (23.8%)	225/1040 (21.6%)	478/2101 (22.8%)
BMI 30.0–40.0 kg/m^2^	124/1061 (11.7%)	128/1040 (12.3%)	252/2101 (12.0%)
Educational level (n (%))			
General secondary school	156/1060 (14.7%)	173/1036 (16.7%)	329/2096 (15.7%)
Intermediate secondary school	454/1060 (42.8%)	430/1036 (41.5%)	884/2096 (42.2%)
High school	450/1060 (42.4%)	433/2096 (41.8%)	883/2096 (42.1%)
Country of birth (n (%))			
Germany	932/1060 (87.9%)	930/1037 (89.7%)	1862/2097 (88.8%)
Others	128/1060 (12.1%)	107/1037 (10.3%)	235/2097 (11.2%)
Nulliparous (n (%))	660/1061 (62.2%)	557/1039 (53.6%)	1217/2100 (58.0%)
Living with a partner (n (%))	1022/1057 (96.7%)	988/1037 (95.3%)	2010/2094 (96.0%)
Full-time employed	568/1047 (54.3%)	514/1031 (49.9%)	1082/2078 (52.1%)

^a^mean ± SD

### Physical activity behaviour

Table 2 shows unadjusted data on the total PA behaviour as well as the PA behaviour categorised by intensity and type. The corresponding adjusted models are depicted in Table 3. In some types and intensities, mean PA level tended to be higher in the C compared to the IV at baseline (T0). Although there seemed to be no major overall difference between IV and C at T1 in unadjusted models, there was evidence of between-group differences in some intensities, types and in total PA when adjusting for pre-pregnancy age, pre-pregnancy BMI, parity and baseline (T0).

**Table 2:** Unadjusted data on the physical activity behaviour of study participants

	**Time point**	**Intervention group**		**Control group**		**Effect size (95% CI)**	***p*** **value**
		**n**	**Mean ± SD**	**n**	**Mean ± SD**		
Total PA							
Total PA	T0	*n* = 975	166.2 ± 65.6	*n* = 935	176.0 ± 75.1	1.18 (−4.26, 6.61)	0.672
	T1	*n* = 906	144.5 ± 59.8	*n* = 908	144.4 ± 67.9		
Time effect	*p* < 0.001			*p* < 0.001			
TALIA	T0	*n* = 978	153.2 ± 65.7	*n* = 942	162.7 ± 75.4	1.62 (−4.12, 7.36)	0.580
	T1	*n* = 909	129.8 ± 61.4	*n* = 911	129.7 ± 69.1		
Time effect	*p* < 0.001			*p* < 0.001			
Intensity							
Sedentary	T0	*n* = 1017	13.0 ± 10.8	*n* = 971	13.0 ± 11.8	−0.44 (−1.65, 0.76)	0.472
	T1	*n* = 953	14.5 ± 12.1	*n* = 939	14.9 ± 12.7		
Time effect	*p* = 0.001			*p* < 0.001			
Light-intensity	T0	*n* = 1002	104.6 ± 39.0	*n* = 965	107.5 ± 41.2	2.13 (−3.33, 7.59)	0.445
	T1	*n* = 937	90.7 ± 41.8	*n* = 926	90.2 ± 42.0		
Time effect	*p* < 0.001			*p* < 0.001			
Moderate-intensity	T0	*n* = 992	47.5 ± 47.3	*n* = 953	53.7 ± 56.0	−0.34 (−3.94, 3.26)	0.853
	T1	*n* = 922	38.3 ± 33.6	*n* = 921	39.2 ± 39.7		
Time effect	*p* < 0.001			*p* < 0.001			
Vigorous-intensity	T0	*n* = 1017	1.4 ± 3.5	*n* = 976	1.5 ± 4.2	0.33 (0.10, 0.55)	0.004
	T1	*n* = 959	0.9 ± 2.5	*n* = 938	0.6 ± 2.2		
Time effect	*p* < 0.001			*p* < 0.001			
Type							
Household activity	T0	*n* = 1005	68.9 ± 55.0	*n* = 962	76.4 ± 60.0	−5.37 (−14.14, 3.39)	0.230
	T1	*n* = 934	69.7 ± 49.6	*n* = 921	75.2 ± 57.0		
Time effect	*p* = 0.564			*p* = 0.127			
Occupational activity	T0	*n* = 775	72.0 ± 42.3	*n* = 739	74.7 ± 49.4	3.93 (−3.34, 11.20)	0.289
	T1	*n* = 450	61.8 ± 36.4	*n* = 407	59.3 ± 40.3		
Time effect	*p* < 0.001			*p* < 0.001			
Sport activity	T0	*n* = 1007	9.9 ± 9.4	*n* = 965	9.6 ± 9.9	2.30 (1.10, 3.50)	< 0.001
	T1	*n* = 938	11.9 ± 9.1	*n* = 931	9.8 ± 9.1		
Time effect	*p* < 0.001			*p* = 0.282			
Transportation activity	T0	*n* = 1006	15.0 ± 12.3	*n* = 973	15.3 ± 13.3	0.60 (−1.49, 2.69)	0.575
	T1	*n* = 944	14.0 ± 11.4	*n* = 938	14.0 ± 12.1		
Time effect	*p* = 0.009			*p* = 0.004			
Inactivity	T0	*n* = 1017	17.6 ± 14.0	*n* = 969	17.6 ± 14.6	−0.29 (−1.67, 1.09)	0.681
	T1	*n* = 951	19.2 ± 14.7	*n* = 935	19.4 ± 14.9		
Time effect	*p* < 0.001			*p* < 0.001			
Meeting PA recommendations (n (%))^1^						OR (95% CI)	
	T0		504/1007 (50.0%)	428/965 (44.4%)			
	T1		597/938 (63.6%)	458/931 (49.2%)		1.84 (1.36, 2.48)	< 0.001

Depicted are mean MET-h/week ± SD; OR: Odds ratio; PA: Physical activity;

T0: Assessment before the end of the 12th week of gestation;

T1: Assessment after the 29th week of gestation;

TALIA: Total Physical Activity of Light Intensity and Above.

^1^Meeting recommendations defined as ≥ 7.5 MET-h/week in category sports activity of moderate-intensity or greater

**Table 3:** Adjusted data on the physical activity behaviour of study participants

	**Time point**	**Intervention group**		**Control group**		**Adjusted effect size**^**1**^ **(95% CI)**	**Adjusted**^**1**^ ***p*** **value**
		**n**	**Mean ± SD**	**n**	**Mean ± SD**		
Total PA							
Total PA	T0	*n* = 975	166.2 ± 65.6	*n* = 935	176.0 ± 75.1	6.00 (4.93, 7.07)	< 0.001
	T1	*n* = 906	144.5 ± 59.8	*n* = 908	144.4 ± 67.9		
Time effect			*p* < 0.001^2^		*p* < 0.001^2^		
TALIA	T0	*n* = 978	153.2 ± 65.7	*n* = 942	162.7 ± 75.4	6.78 (5.64, 7.93)	< 0.001
	T1	*n* = 909	129.8 ± 61.4	*n* = 911	129.7 ± 69.1		
Time effect			*p* < 0.001^2^		*p* < 0.001^2^		
Intensity							
Sedentary	T0	*n* = 1017	13.0 ± 10.8	*n* = 971	13.0 ± 11.8	−0.75 (−1.75, 0.24)	0.137
	T1	*n* = 953	14.5 ± 12.1	*n* = 939	14.9 ± 12.7		
Time effect			*p* = 0.001^2^		*p* < 0.001^2^		
Light-intensity	T0	*n* = 1002	104.6 ± 39.0	*n* = 965	107.5 ± 41.2	4.12 (−0.49, 8.73)	0.080
	T1	*n* = 937	90.7 ± 41.8	*n* = 926	90.2 ± 42.0		
Time effect			*p* < 0.001^2^		*p* < 0.001^2^		
Moderate-intensity	T0	*n* = 992	47.5 ± 47.3	*n* = 953	53.7 ± 56.0	2.39 (0.31, 4.48)	0.024
	T1	*n* = 922	38.3 ± 33.6	*n* = 921	39.2 ± 39.7		
Time effect			*p* < 0.001^2^		*p* < 0.001^2^		
Vigorous-intensity	T0	*n* = 1017	1.4 ± 3.5	*n* = 976	1.5 ± 4.2	0.32 (0.12, 0.51)	0.002
	T1	*n* = 959	0.9 ± 2.5	*n* = 938	0.6 ± 2.2		
Time effect			*p* < 0.001^2^		*p* < 0.001^2^		
Type							
Household activity	T0	*n* = 1005	68.9 ± 55.0	*n* = 962	76.4 ± 60.0	0.78 (−5.79, 7.35)	0.815
	T1	*n* = 934	69.7 ± 49.6	*n* = 921	75.2 ± 57.0		
Time effect			*p* = 0.564^2^		*p* = 0.118^2^		
Occupational activity	T0	*n* = 775	72.0 ± 42.3	*n* = 739	74.7 ± 49.4	1.37 (−1.56, 4.30)	0.360
	T1	*n* = 450	61.8 ± 36.4	*n* = 407	59.3 ± 40.3		
Time effect			*p* < 0.001^2^		*p* < 0.001^2^		
Sport activity	T0	*n* = 1007	9.9 ± 9.4	*n* = 965	9.6 ± 9.9	1.88 (0.95, 2.81)	< 0.001
	T1	*n* = 938	11.9 ± 9.1	*n* = 931	9.8 ± 9.1		
Time effect			*p* < 0.001^2^		*p* = 0.305^2^		
Transportation activity	T0	*n* = 1006	15.0 ± 12.3	*n* = 973	15.3 ± 13.3	0.65 (−1.05, 2.36)	0.454
	T1	*n* = 944	14.0 ± 11.4	*n* = 938	14.0 ± 12.1		
Time effect			*p* = 0.009^2^		*p* = 0.004^2^		
Inactivity	T0	*n* = 1017	17.6 ± 14.0	*n* = 969	17.6 ± 14.6	−0.69 (−1.77, 0.39)	0.208
	T1	*n* = 951	19.2 ± 14.7	*n* = 935	19.4 ± 14.9		
Time effect			*p* < 0.001^2^		*p* < 0.001^2^		
Meeting PA recommendations (n (%))^3^						adjusted OR (95% CI)	
	T0		504/1007 (50.0%)		428/965 (44.4%)	1.69 (1.28, 2.23)	< 0.001
	T1		597/938 (63.6%)		458/931 (49.2%)		

Depicted are mean MET-h/week ± SD; OR: Odds ratio; PA: Physical activity;

T0: Assessment before the end of the 12th week of gestation; T1: Assessment after the 29th week of gestation;

TALIA: Total Physical Activity of Light Intensity and Above.

^1^adjusted for pre-pregnancy age, pre-pregnancy BMI, parity, T0

^2^adjusted for pre-pregnancy age, pre-pregnancy BMI, parity

^3^Meeting recommendations defined as ≥ 7.5 MET-h/week in category sports activity of moderate-intensity or greater

Both total PA (adjusted effect size 6.00 MET-h/week, 95% CI 4.93 to 7.07 MET-h/week; *p* < 0.001) as well as TALIA (adjusted effect size 6.78 MET-h/week, 95% CI 5.64 to 7.93 MET-h/week; *p* < 0.001) differed significantly between groups in late pregnancy. Moreover, groups differed significantly in their level of moderate-intensity activity (adjusted effect size 2.39 MET-h/week, 95% CI 0.31 to 4.48 MET-h/week; *p* = 0.024), their level of vigorous-intensity activity (adjusted effect size 0.32 MET-h/week, 95% CI 0.12 to 0.51 MET-h/week; *p* = 0.002) and their level of sport activity (adjusted effect size 1.88 MET-h/week, 95% CI 0.95 to 2.81 MET-h/week; *p* < 0.001) at T1. In total, 50.0% of participants in the IV and 44.4% in the C met the PA recommendations at T0 and 63.6 and 49.2% at T1, respectively. There was significant evidence of a between-group difference in meeting the PA recommendations at T1 (adjusted OR 1.69, 95% CI 1.28 to 2.23; *p* < 0.001).

The mean MET-h/week in sedentary activity increased significantly in both groups during the course of pregnancy (IV: adjusted *p* = 0.001; C: adjusted *p* < 0.001), as did the level of inactivity (adjusted *p* < 0.001 in both groups). In most categories, women in both groups decreased the PA level significantly from early to late pregnancy. However, women in the IV increased their mean MET-h/week in sport activity over the course of pregnancy (adjusted *p* < 0.001), while no change was observed in the C (adjusted *p* = 0.305).

### Factors influencing prenatal physical activity

Differences in TALIA at T1 according to group assignment were studied in different subgroups (Table 4). There was significant evidence of a difference between IV and C in women with normal weight in late pregnancy (adjusted effect size 6.70, 95% CI 3.99 to 9.41; *p* < 0.001), but not in women with overweight or obesity. Women of older age categories (26–35 years: adjusted effect size 7.25 MET-h/week, 95% CI 4.84 to 9.66 MET-h/week; *p* < 0.001; 36–43 years: adjusted effect size 14.33 MET-h/week, 95% CI 2.97 to 25.68 MET-h/week; *p* = 0.013) and higher educational levels (Intermediate secondary school: adjusted effect size 7.54 MET-h/week, 95% CI 2.05 to 13.03; *p* = 0.007; High school: adjusted effect size 5.61 MET-h/week, 95% CI 3.28 to 7.94 MET-h/week; *p* < 0.001) in the IV differed in their level of TALIA at T1 significantly from women of the C in the corresponding subgroups (Table 4).

**Table 4:** Physical activity behaviour stratified by subgroups in late pregnancy

**TALIA in late pregnancy (T1)**	**Intervention group**		**Control group**		**Adjusted effect size (95% CI)**	**Adjusted** ***p*** **value**
	**n**	**Mean ± SD**	**n**	**Mean ± SD**		
Pre-pregnancy BMI category (n (%))^a^						
BMI 18.5–24.9 kg/m^2^	*n* = 593	129.8 ± 58.3	*n* = 592	127.8 ± 67.3	6.70 (3.99, 9.41)	< 0.001
BMI 25.0–29.9 kg/m^2^	*n* = 219	133.7 ± 68.0	*n* = 201	130.8 ± 65.7	5.49 (−1.93, 12.91)	0.147
BMI 30.0–40.0 kg/m^2^	*n* = 97	120.4 ± 63.3	*n* = 118	137.2 ± 82.8	6.34 (−4.51, 17.18)	0.252
**Age categories**^b^						
18–25 years	*n* = 114	118.5 ± 62.2	*n* = 130	129.2 ± 77.3	−3.26 (−16.40, 9.89)	0.627
26–35 years	*n* = 689	131.4 ± 61.1	*n* = 657	130.9 ± 69.0	7.25 (4.84, 9.66)	< 0.001
36–43 years	*n* = 106	131.5 ± 61.1	*n* = 122	123.4 ± 60.4	14.33 (2.97, 25.68)	0.013
Educational level^c^						
General secondary school	*n* = 124	132.4 ± 68.3	*n* = 141	134.7 ± 85.3	8.98 (−1.27, 19.23)	0.086
Intermediate secondary school	*n* = 394	127.1 ± 65.3	*n* = 388	128.6 ± 69.7	7.54 (2.05, 13.03)	0.007
High school	*n* = 390	131.6 ± 54.7	*n* = 380	128.9 ± 61.5	5.61 (3.28, 7.94)	< 0.001

Depicted are mean MET-h/week ± standard deviation

T0: Assessment before the end of the 12th week of gestation;

T1: Assessment after the 29th week of gestation;

TALIA: Total Physical Activity of Light Intensity and Above.

^a^adjusted for pre-pregnancy age, parity, T0

^b^adjusted for pre-pregnancy BMI, parity, T0

^c^adjusted for pre-pregnancy age, pre-pregnancy BMI, parity, T0

Irrespective of group allocation, educational level at T1 significantly influenced the overall TALIA level but no joint effects of group assignment with either educational level or pre-pregnancy BMI category or age on TALIA were observed at T1 (data not shown).

### Effect of physical activity intensities on GWG

Cohort analysis found no significant evidence of an effect of PA intensities on overall GWG at T0 (Table 5). In late pregnancy, TALIA (*p* = 0.006), light-intensity activity (*p* = 0.002) and vigorous-intensity activity (*p* = 0.014) were negatively associated with overall GWG, whereas a trend towards a slight positive association of sedentary activity and total GWG (*p* = 0.103) was observed (Table 5).

**Table 5:** Effect of activity intensities on overall GWG

	**T0**		**T1**	
**Intensities**	**Adjusted effect size**^**1**^ **(95% CI)**	**Adjusted** ***p*** **value**^1^	**Adjusted effect size**^**1**^ **(95% CI)**	**Adjusted** ***p*** **value**^**1**^
TALIA	0.01 (−0.03, 0.04)	0.609	−0.05 (−0.09, −0.02)	0.006
Sedentary	0.06 (−0.16, 0.28)	0.582	0.17 (−0.03, 0.36)	0.103
Light-intensity	0.01 (−0.05, 0.07)	0.861	−0.09 (−0.15, −0.03)	0.002
Moderate-intensity	0.00 (−0.05, 0.05)	0.904	−0.05 (−0.11, 0.02)	0.186
Vigorous-intensity	−0.36 (−1.00, 0.28)	0.273	−1.34 (−2.41, −0.27)	0.014

Estimated is effect of 10 MET-h/week change in intensities on overall GWG

T0: Assessment before the end of the 12th week of gestation;

T1: Assessment after the 29th week of gestation;

TALIA: Total Physical Activity of Light Intensity and Above;

GWG: Gestational weight gain.

^1^adjusted for pre-pregnancy age, pre-pregnancy BMI, parity, group assignment

## Discussion

The purpose of this secondary analysis was to investigate the impact of a lifestyle intervention programme with basic PA advice in a routine care setting on prenatal PA behaviour. Although the intervention observed no effect on excessive GWG, which was the main outcome of the GeliS trial [42], secondary analyses showed some positive effects of the intervention on intensity and type of reported PA. Significant between-group differences were found in the level of total PA, in TALIA, in moderate-, and vigorous-intensity activities as well as in the level of sport activities. The national and international PA recommendations for pregnant women were more often met in the IV. This highlights the success of the GeliS intervention in improving antenatal PA behaviour. As expected, PA declined over the course of pregnancy, which was equally observed by others [30, 53, 54] and might be explained by an expected increasing discomfort in engaging in PA as pregnancy progressed [55].

Previous antenatal lifestyle interventions differed in their study design, setting, participant characteristics and PA data collection. Therefore, it is difficult to compare findings. However, using the International Physical Activity Questionnaire to estimate antenatal PA behaviour, our pilot trial FeLIPO found no between-group differences, but observed a significant reduction of total PA in the course of pregnancy only in the control [40]. In contrast to findings from the FeLIPO trial, we observed in this study that both groups decreased their total PA level over time. Nevertheless, the IV showed a higher level of vigorous-intensity activities and was even able to increase the level of sport activities, while the latter remained unchanged in the C. Two other large-scaled antenatal RCTs included women with overweight and/or obesity only and used different questionnaires to assess PA [35, 56]. In line with our observations, both trials detected significant between-group differences in the level of total PA in late pregnancy. In LIMIT, these results were mainly explained by significant differences in household activities [56]. However, data on PA intensities are not published. The authors of the UPBEAT trial attributed their observed difference to the fact that participants of the IV spent more time walking compared to the standard care group [35]. In contrast to GeliS, the authors found no differences in moderate- and vigorous-intensity activities [35]. Thus, the between-group differences in the level of moderate- and vigorous-intensity activities in GeliS may be explained in particular by different PA patterns in women with normal weight. This is in line with the observation of significant between-group differences in the level of TALIA at T1 only in the subgroup of women with normal weight.

The LIMIT and the UPBEAT trials placed a stronger emphasis on antenatal PA behaviour by including supervised walking sessions, exercise videos and PA monitoring tools, whereas the GeliS study only provided basic PA advice and distributed leaflets to participants. The question remains whether PA modification should be considered as a critical component of antenatal interventions and whether it has the potential to mitigate several maternal health outcomes. In this context, Simmons et al. (2017) compared the effectiveness of three lifestyle interventions (diet, PA, diet and PA combined) for women with a BMI ≥ 29.0 kg/m^2^ with usual care [57]. The joint intervention, including both diet and PA coaching, had the greatest effect and resulted in substantially lower GWG (− 2.02 kg; 95% CI − 3.58 to − 0.46 kg) and a lower risk for excessive GWG (OR: 2.13; 95% CI 1.05 to 4.33) compared to the usual care group. Despite improvements in the PA and dietary behaviour of participants [47], we found no evidence that the GeliS intervention succeeded in reducing the proportion of women with excessive GWG, although we likewise focused on both lifestyle factors [42]. Nevertheless, we detected significant differences in the level of vigorous-intensity activities between women with and without excessive GWG and trends for differences in TALIA and moderate-intensity activities (data not shown). Furthermore, total GWG was inversely associated with TALIA, light- and vigorous-intensity activities in late pregnancy. This overall effect of PA on GWG is supported by current research. A meta-analysis including RCTs which only implemented a PA intervention reported beneficial effects on overall GWG (*p* < 0.001) in women of all BMI categories [58]. Correspondingly, a recently published meta-analysis, including studies with normal weight women only, concluded that exercise during pregnancy can reduce GWG (mean difference = − 1.61 kg, 95% CI − 1.99 to − 1.22 kg) [20] and highlights the need for large-scale interventions and for including normal weight women. Both demands were met within the GeliS trial. Aside from decreasing the risk for excessive GWG, research found that prenatal PA influences a woman’s overall health status due to beneficial effects on physiological and psychological well-being and decreases the risk for pregnancy-induced complications [18]. Drawing from the evidence presented herein, we suggest that pregnant women should be encouraged to engage in an active lifestyle according to the ACOG recommendations.

In the GeliS study, women in the IV (63.6%) were more likely to meet the national and international PA recommendations [43, 44] in late pregnancy than women in the C (49.2%). However, compared to other observations, the adherence of both groups to PA recommendations is relatively high. The percentage of pregnant women meeting the PA recommendations of the ACOG [44], depending on different thresholds, was estimated to range between 12.7 and 45.0% [28]. On the one hand, the discrepancy with GeliS observations might be explained by self- and over-reporting, leading to a higher percentage of women that were found to meet the recommendations. On the other hand, estimating whether or not women adhere to the PA recommendations by means of the PPAQ is prone to error, although it was similarly done by others [51] and recommended by the developer of the questionnaire (personal communication).

There are further limitations of this secondary analysis. First, we observed differences in the baseline PA level between women in the IV and C groups. In order to overcome this limitation and to more accurately assess the intervention effect on PA behaviour, we included baseline PA level (T0) as a covariate. This most likely explains why we found significant evidence for between-group differences in adjusted but not in unadjusted models in late pregnancy. We reported PA behaviour at two different time points in pregnancy, before the end of the 12th week and after the 29th week of gestation. Assessing PA behaviour shortly before delivery, would have given further insights into the PA decline over the entire course of pregnancy. We assessed PA by means of a frequency questionnaire, which could lead to inaccuracies as physical activity questionnaires in general are known to have limited reliability and validity [59]. Nevertheless, the questionnaire we administered was extensively validated [48] and recommended for PA assessments in pregnancy [60]. We slightly adapted it to German habits and are not aware of its implication on the overall validity, although we do not anticipate differences in PA levels compared to using the original PPAQ. It is important to consider, that the self-administration of the PPAQ might have introduced selective bias and might have influenced observed results. The GeliS study was performed outside an academic setting. Interviewer-administered PPAQs or any other type of PA assessment were not feasible. Thus, a comparison between PA data of GeliS and studies who applied interviewer-administered PPAQs [51, 61] is more challenging. As with any self-reported activity questionnaire, we face the problem that self-reports rely on the subjective estimation of participants and on their ability to remember their physical activity level and type of performed sports for the past 4 weeks. Moreover, self-reports are susceptible to over- and under-reporting [62], which cannot completely be excluded within the presented data. For instance, we found higher levels of TALIA and more outliers in the subgroup of women with overweight, and in particular in women with obesity in the C group (data not shown). However, we intended to minimise this influence by clearly defining over-reporting, a priori, as described in the Methods part. To this end, reported unrealistic activity levels were not included in the analyses. The overall prevalence of normal weight, overweight and obesity in the GeliS cohort differs from the general population of women of childbearing age in Germany, which makes our findings difficult to generalise [63]. Furthermore, educational level was found to significantly influence TALIA in late pregnancy but was not controlled for in our adjusted model. However, we observed neither group differences in educational level nor an interaction with group allocation. Therefore, we can conclude that educational level influences prenatal PA behaviour without distorting between-group observations. Finally, we did not include dietary intake and in particular dietary modification as covariate and are aware that this shortcoming might lead to a slightly biased estimation of the effect of prenatal PA on overall GWG [47]. We acknowledge that a more detailed PA intervention, such as counselling given by PA experts, integrating behavioural change strategies, supervised PA classes or digital activity trackers and smartphone applications might have strengthened the effect of the intervention on PA and could lead to an impact on GWG. An evaluation of incorporated behaviour change techniques, by applying behaviour change taxonomies from the beginning on, would provide valuable details about the quality of the intervention.

Apart from these limitations, the secondary findings presented herein have several strengths that are worth noting. By pooling both groups to form one cohort, it was possible to assess the influence of different PA intensities on overall GWG. To our knowledge, there is no other trial that addressed this effect in such detail. In addition, we were able to estimate the PA behaviour, including type and intensity of PA, of women in all BMI categories. Moreover, we could demonstrate PA behaviour patterns of women who had received basic PA advice given by trained counsellors in the routine care setting. To the best of our knowledge, there is no other trial to-date which was conducted on a large scale in a routine care setting that showed such comprehensive findings on the impact of a lifestyle intervention on prenatal PA behaviour.

Considering the public health approach of this study, providing only simple recommendations was a feasible and a realistic way of motivating pregnant women to engage in PA and to maintain an active lifestyle during the course of pregnancy. However, future approaches could implement some of the above strategies to examine if complementary methods of self-monitoring coupled with expert instruction may exert a more pronounced change on prenatal lifestyle, and ultimately, on GWG.

## Conclusion

This secondary analysis demonstrates that the GeliS intervention was moderately effective in improving the antenatal PA behaviour in a routine care setting. As there was no difference between groups in the proportion of women with excessive weight gain, a moderate change in PA and dietary behaviour alone might not be sufficient to have a significant impact on overall GWG. Subsequent analyses of the GeliS mother-child cohort might reveal the effect of antenatal PA behaviour on other maternal and offspring parameters, with a special focus on its long-term impact on maternal and infant health.

## Data Availability

The datasets used and analysed during the current study are available from the corresponding author on reasonable request.

## Abbreviations

ACOG: American College of Obstetrics and Gynecology
BMI: Body-Mass-Index
C: Control group
CI: Confidence interval
GDM: Gestational diabetes mellitus
GEE: Generalised estimating equations
GeliS: “Gesund leben in der Schwangerschaft”/ “healthy living in pregnancy“
GWG: Gestational weight gain
IOM: Institute of Medicine
IV: Intervention group
MET: Metabolic equivalent of task
OR: Odds ratio
PA: Physical activity
PPAQ: Pregnancy Physical Activity Questionnaire
RCT: Randomised-controlled trial
SD: Standard deviation
TALIA: Total Physical Activity of Light Intensity and Above
