# Correction To: Effects of a lifestyle intervention in routine care on prenatal physical activity – findings from the cluster-randomised GeliS trial

**DOI:** 10.1186/s12909-019-1889-z

**Published:** 2019-12-24

**Authors:** Julia Hoffmann, Julia Günther, Kristina Geyer, Lynne Stecher, Kathrin Rauh, Julia Kunath, Dorothy Meyer, Christina Sitzberger, Monika Spies, Eva Rosenfeld, Luzia Kick, Renate Oberhoffer, Hans Hauner

**Affiliations:** 1 Else Kröner-Fresenius-Centre for Nutritional Medicine, Klinikum rechts der Isar, Technical University of Munich, Georg-Brauchle-Ring 62, 80992 Munich, Germany; 2 Competence Centre for Nutrition (KErn), Am Gereuth 4, 85354 Freising, Germany; 3 Institute of Preventive Pediatrics, Technical University Munich, Georg-Brauchle-Ring 62, 80992 Munich, Germany; 4 Department of Pediatric Cardiology and Congenital Heart Defects, German Heart Centre, Lazarettstraße 36, 80636 Munich, Germany

**Correction to: BMC Pregnancy Childbirth (2019) 19:414**

**https://doi.org/10.1186/s12884-019-2553-7**

Following publication of the original article [1], the author notified us about incorrectly formatted of **Table 2** and **Table 3**. Also, about the incorrectly displayed of the values and the corresponding unit. The original version of this article was revised.

The correct format of the tables is presented below.

**Table 2**

- The time effect *p*-value (11 changes required) should be placed below the corresponding mean±SD-values. The values are always aligned to the right side of the columns.
- The effect size-values and *p*-values account should be for the time point T1 only. The effect sizes (11 changes required) and *p*-values (11 changes required) values should be placed at the same line as “T1”.

**Table 3**

- The effect size-values and *p*-values account should be for the time point T1 only. The effect sizes (12 changes required) and *p*-values (12 changes required) values should to be placed at the same line as “T1”.

The visibility of the tables was also improved.

The values and the corresponding units should be displayed in one line.
